# Impact of intracellular ion channels on cancer development and progression

**DOI:** 10.1007/s00249-016-1143-0

**Published:** 2016-06-11

**Authors:** Roberta Peruzzo, Lucia Biasutto, Ildikò Szabò, Luigi Leanza

**Affiliations:** 1 Department of Biology, University of Padua, Padua, Italy; 2 CNR Institute of Neuroscience, Padua, Italy; 3 Department of Biomedical Sciences, University of Padua, Padua, Italy

**Keywords:** Ion channels, Cancer, Mitochondria, Cancer cell metabolism, Organelles

## Abstract

Cancer research is nowadays focused on the identification of possible new targets in order to try to develop new drugs for curing untreatable tumors. Ion channels have emerged as “oncogenic” proteins, since they have an aberrant expression in cancers compared to normal tissues and contribute to several hallmarks of cancer, such as metabolic re-programming, limitless proliferative potential, apoptosis-resistance, stimulation of neo-angiogenesis as well as cell migration and invasiveness. In recent years, not only the plasma membrane but also intracellular channels and transporters have arisen as oncological targets and were proposed to be associated with tumorigenesis. Therefore, the research is currently focusing on understanding the possible role of intracellular ion channels in cancer development and progression on one hand and, on the other, on developing new possible drugs able to modulate the expression and/or activity of these channels. In a few cases, the efficacy of channel-targeting drugs in reducing tumors has already been demonstrated in vivo in preclinical mouse models.

## Introduction

**Fig. 1:**
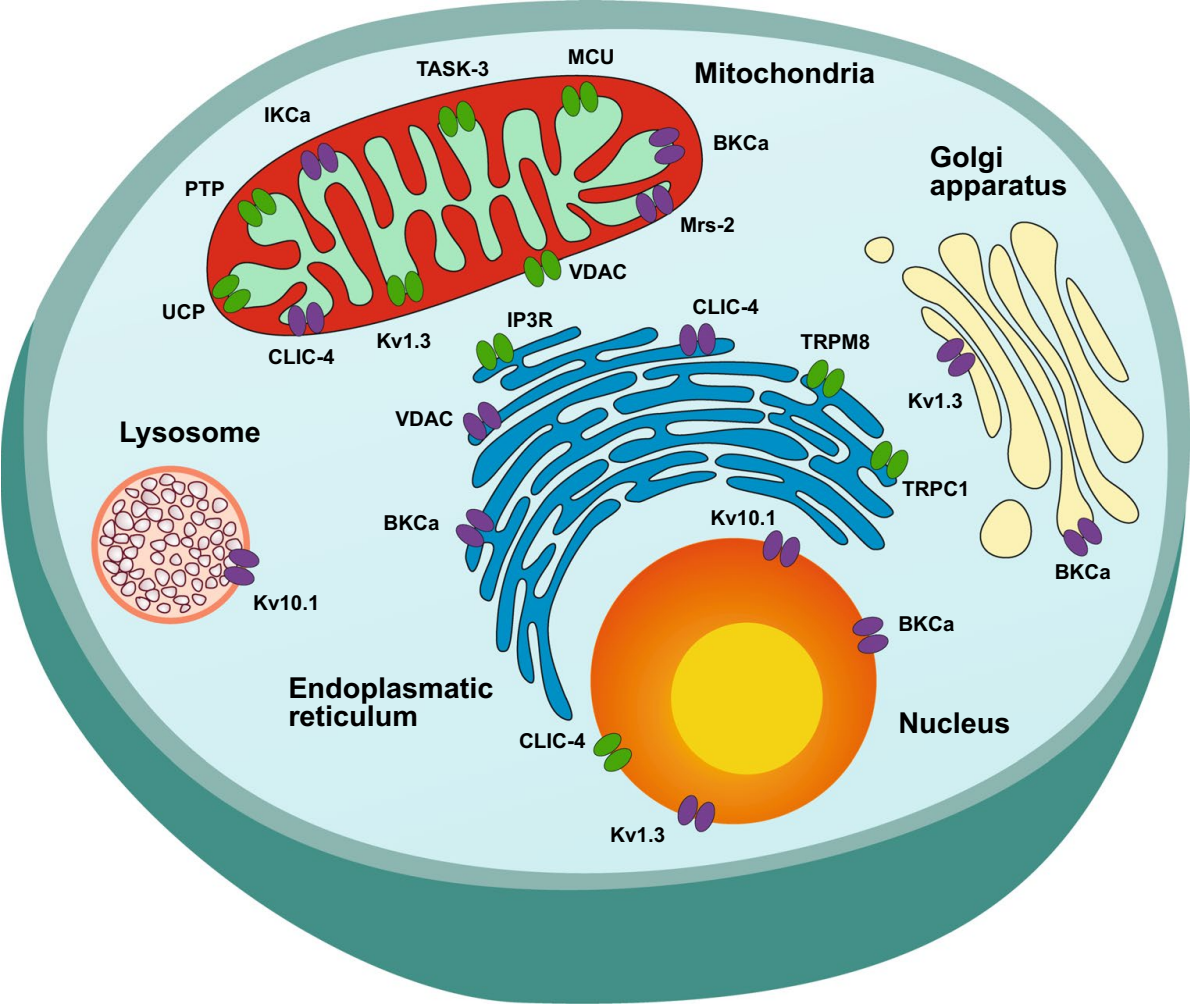
Intracellular ion channels are expressed in several subcellular compartments, such as the nucleus, endoplasmic reticulum, Golgi apparatus, lysosome and mitochondria. Ion channels, whose role in cancer development and/or progression has been demonstrated, are stained in *green*

In the last decades, cancer research has been focused on identifying novel targets for therapy, especially on trying to develop new strategies to fight untreatable tumors. Almost 20 years ago, ion channels were proposed as potential targets: they are often aberrantly expressed in cancer tissues compared to healthy ones, and, importantly, there are many pharmacological tools already available to manipulate them. Ion channels have been demonstrated to contribute to the acquirement of several hallmarks of cancer cells (Hanahan and Weinberg 2011): metabolic re-programming (e.g., Andersen et al. 2014; Leanza et al. 2013a, 2014b), limitless proliferative potential (Urrego et al. 2014; Pardo and Stühmer 2014), apoptosis resistance (Wang 2004; Hoffmann and Lambert 2014), stimulation of neo-angiogenesis (Munaron 2015) as well as cell migration and invasiveness (Litan and Langhans 2015; Djamgoz and Onkal 2013). In recent years, not only plasma membrane channels but also intracellular ones have been related to the control of some typical features in cancer cells (Leanza et al. 2013a). Inside the cell, ion channels are expressed in several organelles (Xu et al. 2015): mitochondria, endoplasmic reticulum, nucleus, lysosome, Golgi apparatus, peroxisomes and endosomes. Most of the identified “oncogenic” intracellular channels are located in the outer or inner mitochondrial membrane (OMM and IMM), since this organelle is a key point of control of important hallmarks of cancer, such as ATP-linked metabolism and apoptotic cell death. Another relevant intracellular location is the endoplasmic reticulum (ER): ion channels and transporters of the ER modulate the cytosolic Ca^2+^ concentration by controlling the uptake and release of calcium from this intracellular store and thus impact several cellular pathways implicated in the determination of cell fate as well in cell metabolism. Figure 1 summarizes the intracellular channels that have been linked to tumorigenesis or have been identified as possible targets.

The relevance of studying oncogenic channels is underlined by recent studies demonstrating that their pharmacological modulation can reduce tumor growth even in vivo in preclinical models of brain, lung, stomach, colon, blood, cervix, bladder, skin, prostate, breast, esophagus and oral cavity cancers (Leanza et al. 2015a).

In this review, we discuss intracellular channels whose altered expression has been related to cancer development or progression. Furthermore, we mention cases where pharmacological modulation of these channels points toward a possibility of exploiting them for treatment.

## Mitochondrial channels

### Voltage-dependent anion channel (VDAC)

The identification of voltage-dependent anion channels (VDACs) succeeded using electrophysiology and electron microscopy, since it was demonstrated that the outer mitochondrial membrane (OMM) leak was due to unique anion-selective channels, which transport cations, such as Ca^2+^, as well as many charged and non-charged metabolites, with selectivity for anions over cations being modified by membrane voltage (Colombini and Mannella 2012).

VDAC mediates metabolic cross-talk between the mitochondria and the rest of the cell (Shoshan-Barmatz et al. 2010; Shoshan-Barmatz and Mizrachi 2012; Reddy 2013), is a key player in the regulation of mitochondria-mediated apoptosis and is implicated in cancer and neurodegenerative disorders (Shoshan-Barmatz et al. 2010; Shoshan-Barmatz and Golan 2012; Shoshan-Barmatz and Mizrachi 2012; Reddy 2013). In mammals, three versions of VDAC have been identified, VDAC1, VDAC2 and VDAC3 (Blachly-Dyson and Forte 2001), which have similar molecular weights (30–35 kDa), share approximately 70 % identity and show structural and functional homology. The three isoforms are nearly ubiquitous in all tissue types, with VDAC2 and VDAC3 expression being lower than VDAC1 (Messina et al. 2012). Recombinant VDAC1 and VDAC2 form channels upon reconstitution into a lipid bilayer (BLM) (Xu et al. 1999). Recently, also hVDAC3 was shown to give rise to channel activity in BLM displaying however a very small conductance (100 pS) in contrast to VDAC1 (4 nS in 1 M KCl) (Checchetto et al. 2014). VDAC3 has recently been proposed to act as a redox sensor in the cells, given that its function depends on the redox state of critical cysteine residues that face the intermembrane space (Reina et al. 2016). Interestingly, there are many cases where VDACs have been mutated in various cancer tissues, although the relevance of these mutations in the context of tumor progression is still unclear (Naghdi and Hajnóczky 2016).

Besides its presence in the OMM, VDAC1 has also been localized by immunofluorescence, flow cytometry and EM immunogold labeling in other cell compartments, such as the plasmamembrane (De Pinto et al. 2010), sarcoplasmic reticulum of skeletal muscles (Shoshan-Barmatz et al. 1996), endoplasmic reticulum (ER) of rat cerebellum (Shoshan-Barmatz et al. 2004) and caveolae and caveolae-like domains. However, its function in compartments other than the mitochondria is less clear.

VDAC1 is overexpressed in many cancer types (Shoshan-Barmatz et al. 2015). Thus, VDAC1 is an excellent target for anti-cancer therapy since thanks to its observed low genetic variability between patients, the chances that tumors will develop resistance to VDAC1-based drugs by acquiring mutations in VDAC1 are low. The role of VDAC1 in cellular metabolism is crucial, since it serves as the main interface between mitochondrial and cellular metabolisms by allowing a two-way traffic: it mediates the entry of metabolites including pyruvate, malate, succinate, ADP and NADH into mitochondria and the exit of newly formed molecules, such as hemes and ATP (Shoshan-Barmatz et al. 2015). Furthermore, VDAC1 is involved in the regulation of apoptosis, thanks to its role in the release of apoptosis-inducing proteins from the mitochondria to the cytosol (Shoshan-Barmatz et al. 2015).

Importantly, downregulation of VDAC1 expression resulted in a reduced metabolite exchange between mitochondria and the cytosol, inhibited cell growth (Abu-Hamad et al. 2006) and prevented cell death induced by cisplatin (Tajeddine et al. 2008) and endostatin (Yuan et al. 2008). On the contrary, overexpression of VDAC1 was found to induce apoptotic cell death following apoptotic stimuli (Godbole et al. 2003; Zaid et al. 2005; Ghosh et al. 2007; Lu et al. 2007; Abu-Hamad et al. 2008).

The metabolic advantage given by VDAC1 overexpression to cancer cells is due to the fact that it presents binding sites for overexpressed Hexokinase, allowing direct transport of mitochondrial ATP for glucose phosphorylation, thus increasing the glycolytic rate, a characteristic of cancer cells (i.e., the Warburg effect). In addition, VDAC1 also binds Bcl-2 and Bcl-xL, anti-apoptotic proteins expressed in many cancer cells, and impacts their cell-saving function (Shoshan-Barmatz et al. 2015). Bcl-xL was shown to interact with VDAC1 to inhibit apoptosis promoted by mitochondrial Ca^2+^ uptake (Roy et al. 2009; Monaco et al. 2015). Similary, binding of Bcl-2 to VDAC1 inhibits cytochrome c release and apoptosis (Abu-Hamad et al. 2009). In contrast, other studies showed that VDAC1 interacts with Bax to exert a pro-apoptotic effect (Shimizu et al. 2000; Shi et al. 2003).

Several studies have shown differences in the expression of VDAC between cancer cell lines and tissues with respect to the normal counterparts: high VDAC1 levels were observed in hepatoma, sarcomatous alterations (Yoo et al. 2009), non-small-cell lung cancer (NSCLC) cells (Brahimi-Horn et al. 2012), gastric cancer cells (Bai et al. 2011) and different cancer types, such as thyroid, lung, cervix, ovary, pancreas, melanoma and glioblastoma cancers. A several-fold increase in VDAC1 expression was shown also in peripheral blood mononuclear cells from chronic lymphocytic leukemia (CLL) patients (Shoshan-Barmatz et al. 2015). In addition, in melanoma and prostate cancer cell lines, a positive correlation between levels of VDAC1 expression and the release of cytochrome c by G3139 was demonstrated (Lai et al. 2006). VDAC1 expression was also correlated with tumor progression and sensitivity to chemotherapy (Simamura et al. 2008; Pernemalm et al. 2013), whereas gene expression or proteomic analysis of NSCLC or primary lung adenocarcinoma patients revealed high VDAC1 expression levels as a predictor of poor outcome (Grills et al. 2011; Pernemalm et al. 2013). Thus, determination of VDAC1 expression levels can be useful as a molecular biomarker to predict cancer development and treatment efficacy.

Several drugs have been shown to interact with VDAC1 and affect its ability to bind hexokinase and Bcl-2 family member proteins (for a recent review, see, e.g., Szabò and Zoratti 2014): erastin (Yagoda et al. 2007), oblimersen (G3139) (Advani et al. 2011; Pro et al. 2008; Rom et al. 2009; Lai et al. 2006), avicins (Haridas et al. 2005; Gaikwad et al. 2005), cisplatin (Yang et al. 2006; Castagna et al. 2004), endostatin (Yuan et al. 2008) and methyl jasmonate (MJ) (Raviv et al. 2013).

Unfortunately, most of these drugs have pleiotropic effects so that their specificity for VDAC1 is not guaranteed. Indeed, a more specific approach involving specific screening would be required to discover possible novel VDAC1 interactors. In this respect, the design of cell-penetrating VDAC1-based peptides (Shoshan-Barmatz et al. 2015) that impair the interaction between the channel and metabolic regulators and thus impact energy homeostasis and minimize the self-defense mechanisms of cancer cells represents a novel and innovative strategy.

Besides VDAC1, VDAC2 also displays cancer-related features. For example, it is also highly expressed in cancer lines and tissues, and the main target of erastin (see above) is VDAC2. VDAC2 seems to have unique, non-redundant fundamental functions in development and survival, possibly by impacting the mitochondrial import and OMM-permeabilizing function of Bak pro-apoptotic Bcl-2 family proteins, although VDAC2′s role as a promoter or inhibitor of death is still debated (Naghdi and Hajnóczky 2016).

### Permeability transition pore (PTP)

The permeability transition pore (PTP) is a voltage- and Ca^2+^-dependent high-conductance channel (1.3 nS in 150 mM KCl) (Zoratti and Szabò 1995) that is able to dramatically increase in the permeability of the IMM to solutes with molecular mass up to 1500 Da (Bernardi et al. 2006). Indeed, a series of events occurring following PTP opening, including mitochondrial depolarization, generation of reactive oxygen species (ROS), release of mitochondrial Ca^2+^ and swelling of mitochondria leading to breaches in the OMM that induce the release of intermembrane proteins, promotes this channel as a promising oncological target (Rasola and Bernardi 2014). Clearly, long-lasting opening of PTP compromises mitochondrial function and leads to various forms of cell death (apoptosis, necrosis and necroptosis). The key characteristics of PTP are: inhibition by CsA, adenine nucleotides, Mg^2+^, acidic pH and reducing agents, induction by Ca^2+^ and voltage sensitivity (Bernardi et al. 1992; Kinnally et al. 1989; Petronilli et al. 1989, 1991, 1992).

Recently, it has been proposed that the PTP is formed by dimers of the F_O_F_1_ ATP synthase (Giorgio et al. 2013) or by the c-ring of the ATP-producing machinery (Alavian et al. 2014). It has recently been demonstrated that purified ATP synthase from various organisms forms a channel that is similar to the PTP channel and that ablation of e and g ATP synthase subunits of the yeast ATP synthase, which seems to be required for dimer formation, leads to a marked resistance to PTP opening (Carraro et al. 2014). Nevertheless, the molecular identity of the PTP is still debated. A recent work identified mitochondrial spastic paraplegia 7 (SPG7), a nuclear-encoded mitochondrial metalloprotease (m-AAA) that interacts with cyclophilin D and VDAC1 at the IMM and OMM contact site and with a paraplegin-like protein AFG3L2 in the IMM, as an essential component of PTP (Shanmughapriya et al. 2015, but see also Bernardi and Forte 2015). Interestingly, downregulation of SPG7 expression, however only a minor one, can be observed in different cancer tissues (see Table 1): we performed an in silico analysis based on the Oncomine^®^ Research Edition, a web application that integrates and unifies high-throughput cancer-profiling data across a large volume of cancer types, subtypes and experiments so that target expression can be assessed online, to compare expression of a given protein in several cancers compared to normal tissues.

**Table 1:** Oncomine analysis of *SPG7* gene in several human cancer tissues. Here are reported only tumors in which gene expression was changed at least twice compare to normal tissues

Gene	Tissue	Tumor	C vs. N	References
SPG7	Sarcoma	Pleomorphic liposarcoma	−2.502	Detwiller et al. (2005)
	Ovary	Ovarian serous surface papillary carcinoma	−2.089	Welsh et al. (2001)
	Lung	Small cell lung carcinoma	−4.506	Bhattacharjee et al. (2001)
	Kidney	Renal Wilms' tumor	−2.232	Yusenko et al. (2009)
		Renal oncocytoma	−2.735	Yusenko et al. (2009)
	Breast	Invasive breast carcinoma	−2.278	Finak et al. (2008)

As mentioned above, PTP opening induces apoptosis by massive Ca^2+^ release into the cytosol and by impairment of mitochondrial function. The resistance to cell death induction under stress conditions is a key feature of cell progression to malignancy (Hanahan and Weinberg 2000, 2011), and, indeed, some chemotherapeutics are designed to selectively reactivate apoptosis in cancer cells. PTP inhibition is an important adaptation mechanism that acts as a tumor-enhancer event in the model of hepatocarcinogenesis triggered by 2-acetylaminofluorene in rats (Klohn et al. 2003).

Therefore, comprehension of the PTP structure and regulation in cancer progression as well as identification of selective PTP activators is very important to develop anti-neoplastic strategies. However, it has to be mentioned, that the possibility of a selective action on ATP synthase only in cancer cells seems unlikely. Nevertheless, several compounds that open the PTP are under scrutiny as potential chemotherapeutics. Many of them, such as the plant-derived alkaloid berberine (Zhang et al. 2014), the plant hormone methyl jasmonate (Raviv et al. 2013), the monocyclic sesquiterpene alcohol-bisabolol (Cavalieri et al. 2009), the naphtho-quinone shikonin (Han et al. 2007), the triterpenoid betulinic acid (Lena et al. 2009), the constituent of turmeric powder curcumin (Qiu et al. 2014), the polyphenolic compounds resveratrol (Ma et al. 2007) and honokiol (Li et al. 2007b), are natural compounds that have been tested on tumor cell lines and in vivo in preclinical animal models, and some of them are currently undergoing clinical or pre-clinical trials (Leanza et al. 2014b; Suh et al. 2013).

Most of the above-mentioned agents display pro-apoptoptic and anti-neoplastic effects because they induce PTP opening due to increased oxidative stress. Cancer cells exhibit elevated ROS levels, which are produced by both by changes in their metabolism as well as exposure to inappropriate oxygen concentrations (Grek and Tew 2010). This increased level of ROS must be kept under tight control by enhancing antioxidant defenses (De Nicola et al. 2011) in order to avoid the ROS’ damaging effects on several cellular structures, especially in the early tumorigenic phases (Cairns et al. 2011). Under these conditions, the cellular fate is the result of a balance between ROS generation and scavenging, and cancer cells are more vulnerable than normal ones to further oxidative insults. Thus, drugs that act on oxidative damage may represent a strategy for selectively targeting cancer cells (Gorrini et al. 2013). Since PTP induction causes cell death by oxidative stress (Rasola and Bernardi 2011), several pro-oxidant agents that are able to open PTP lead on one hand to an increase in intracellular Ca^2+^ release (due to loss of impermeability) and on the other hand to inhibition of ATP-dependent Ca^2+^ extrusion from the plasmamembrane (due to membrane potential loss and decreased ATP synthesis) (Camello-Almaraz et al. 2006). Indeed, mitochondria are the most important sites for ROS production. ROS produced by the respiratory chain complexes can be exported into the cytosol where they can activate the ER-located IP3 and ryanodine receptors, which can release Ca^2+^ from this intracellular store. In turn, this Ca^2+^ will be (partially) buffered by the MCU. Intra-mitochondrial calcium activates synthesis of reduced substrates (NADH) by metabolic pathways and accelerates the electron transport chain, increasing ROS production, which in turn facilitates Ca^2+^ release by sensitization of IP3R and RyR (Camello-Almaraz et al. 2006). Therefore, strategies to elicit PTP opening can be envisioned as promising anti-neoplastic approaches, even if the possibility of side effects, e.g., on the nervous system or cardiac tissues, must be carefully considered (Leanza et al. 2014b). Conversely, CsA inhibition of the PTP promotes skin cancer in transplant patients, highlighting the key role of PTP inhibition in tumor development (Norman et al. 2010).

Interestingly, overexpression of a serine protease inhibitor of the serpin family, called SERPINB3 (SB3), causes an antioxidant defense mechanism in cancer cells: SB3 locates inside the mitochondria where it inhibits respiratory complex I, thus blocking ROS generation following chemotherapeutic treatment and protecting cells from PTP opening (Ciscato et al. 2014). Further links among PTP induction, ROS generation and respiratory complexes came from studies on the serine/threonine kinase GSK-3, a kinase involved in a variety of biological processes. GSK-3 is constitutively active and exerts an inhibitory effect on its targets and can be itself regulated by serine or tyrosine phosphorylation (Jope and Johnson 2004; Jope et al. 2007). A fraction of the enzyme, called mGSK-3, is located inside in the mitochondria (Chiara and Rasola 2013), where it down-modulates both the Krebs cycle (Hoshi et al. 1996) and oxidative phosphorylation (King et al. 2008), thus constituting an integration point for targets acting on PTP (Juhaszova et al. 2009; Miura and Miki 2009).

In addition, several lines of evidence connect hexokinase II to the PTP, and, for example, it has been shown that detachment of hexokinase II from mitochondria by a selective peptide induces PTP opening triggered apoptosis in several tumor cell models independently of VDAC (Chiara et al. 2008).

In general, mitochondrial hexokinase II is a good target in anti-cancer strategies, since it is required for tumor initiation and maintenance in mouse models of K-Ras-driven lung cancer, as its ablation in conditional knockout mice inhibits tumor growth without side effects (Patra et al. 2013). However, further studies are needed to better understand whether all compounds that target mitochondrial hexokinase II can lead to PTP opening.

Recently, it has been proposed that PTP regulation of tumor cells can be regulated also by molecular chaperones. In particular, the oncogenic TRAP1 was shown to interact with succinate dehydrogenase (SDH), inhibiting succinate oxidation and inducing a pseudohypoxic response (i.e., HIF1 activation in normoxic conditions) (Sciacovelli et al. 2013), but also by shielding tumor cells from ROS-induced PTP opening and death. A decreased TRAP1 expression level was observed in some high-grade tumors. Furthermore, TRAP1 and also the mitochondrial pools of Hsp90 and Hsp60 were reported to interact with CyPD and prevent its ability to induce the pore opening in tumor cell models (Kang et al. 2007; Ghosh et al. 2010). Further work is required to dissect the mode of action of each of these molecular chaperones on the PTP. In summary, PTP is certainly one of the most promising intracellular channels as an oncological target along with VDACs.

### The mitochondrial calcium uniporter (MCU)

Mitochondria act as intracellular conductors of intracellular Ca^2+^ regulation, shaping, remodeling, relaying and decoding Ca^2+^ signals, because of their ability to rapidly and transiently accumulate Ca^2+^ (Drago et al. 2012). In animal cells mitochondria were the first intracellular organelles to be associated with Ca^2+^ handling, and well before the identification of the MCUC components, their ability to rapidly sense Ca^2+^ signals and to act as localized buffers with a high capacity in the proximity of PM and ER Ca^2+^ channels/transporters has been proven (Rizzuto et al. 2012). Indeed, mitochondrial Ca^2+^ uptake, by impacting on local calcium concentrations and on the calcium-mediated feedback mechanism known to modulate the activity of Ca^2+^ channels, influences the frequency and amplitude of cytosolic calcium signals. For example, calcium flux across both the PM/ER-located calcium release induced calcium channel CRAC (Orai1/Stim1) and ER-located inositol-1,4,5-trisphosphate receptor is influenced by the physical vicinity of mitochondria. This proximity, sustained by specific contacts, the so-called MAMs (mitochondria-associated membranes) via chaperones such as sigma receptor 1, in turn sets the extent and duration of the mitochondrial calcium increase. In addition, especially in large cells, recruitment of mitochondria to specific regions seems to be important for constraining Ca^2+^ signals to defined cell domains. As a result, mitochondrial calcium uptake has been shown at least *in vitro* to govern numerous patho-physiological processes ranging from insulin secretion, neuronal excitotoxicity and cardiomyocyte function to tumorigenesis. The reader is advised to consult excellent, recent reviews on this topic (e.g., Rizzuto et al. 2012; Foskett and Philipson 2015).

The mitochondrial calcium uniporter MCU, which mediates uptake of this ion into the mitochondria, actively sequesters cytosolic calcium. Significant advances in identifying the molecular players of the mitochondrial Ca^2+^-handling machinery have been achieved only during the last decade. The finding that a highly Ca^2+^ selective ion channel, displaying a very small conductance of only 5 pS in 100 mM Ca^2+^*in vitro* recapitulated the key characteristics observed for the mammalian mitochondrial uniporter in classical bioenergetic experiments, represented a milestone toward the molecular identification of the uniporter (Kirichok et al. 2004). The MitoCarta database, containing more than 1000 mitochondrial proteins (Pagliarini et al. 2008), then provided the basis for the identification of several mitochondrial calcium uniporter complex (MCUC) components in mammals, including the central pore-forming protein MCU (mitochondrial calcium uniporter; De Stefani et al. 2011; Baughman et al. 2011). At the current stage, MCUC appears to include at least of the pore-forming protein MCU, an MCU paralog (MCUb), the essential MCU regulator (EMRE), the regulatory MICU proteins and, possibly, the mitochondrial calcium uniport regulator 1 (MCUR1) in mammals (De Stefani et al. 2015), unlike in other systems containing a more simplified MCUC (Wagner et al. 2016). This complex composition in mammals may be the reason why it is still unresolved how matrix Ca^2+^ transients are shaped in vivo. What is known from experiments in cells is that Ca^2+^ elevations in the mitochondrial matrix stimulate respiration and ATP synthesis (Denton 2009). Ca^2+^ overload, by contrast, can trigger cell death (Duchen 2000). Increased biosynthesis rates of ATP rely on the activation of three mitochondrial dehydrogenases by Ca^2+^ (McCormack et al. 1990). Pyruvate dehydrogenase (PDH) (Denton et al. 1972), NAD-isocitrate dehydrogenase (NAD-ICDH) (Denton et al. 1978) and oxoglutarate dehydrogenase (OGDH) (McCormack and Denton 1979) are activated by physiologically relevant Ca^2+^ concentrations (100 nM and 1 µM) in mitochondria isolated from mammalian tissues (Denton and McCormack 1980; Denton et al. 1980), and phosphorylation of PDH is thought to be modulated by the calcium-sensitive phosphatase PDP1. Ca^2+^ elevations in intact cells result in NAD(P) reduction (Duchen 1992, Pralong et al. 1992), supporting a central role for Ca^2+^ -dependent regulation of mitochondrial metabolism. PDH activity is regulated through reversible, calcium-dependent phosphorylation (Holness and Sugden 2003; Tovar-Mendez et al. 2003). The mitochondrial [Ca^2+^] increase evoked by a cytosolic [Ca^2+^] rise leads to an enhanced oxidative phosphorylation as well and boosts ATP production. Knock-out of the MCUC regulator MICU1 that results in an increased resting state level of Ca^2+^ in the mitochondrial matrix accordingly alters the PDH phosphorylation state in cultured cells (Mallilankaraman et al. 2012). In addition, lower levels of basal matrix calcium in the *MCU*^−*/*−^ mice led to markedly increased levels of PDH phosphorylation in these animals (Pan et al. 2013), although the animal model used in this study is debated especially in view of the modest phenotype and of the fact that viable mice could be obtained only in a mixed genetic background (see, e.g., Pendin et al. 2014). The consensus view is that conditional and inducible, tissue-specific knockout models, as well as viral-based gene-delivery systems, will be needed to conclusively assess the real physiological impact of mitochondrial calcium homeostasis in vivo.

A few recent in vivo studies in fact demonstrate that mitochondrial calcium homeostasis is crucial for regulation of metabolism and its alterations are linked to pathologies. Genetic manipulation of MCU in lower organisms such as *Zebrafish* (Prudent et al. 2013) and *Trypanosome brucei* (Huang et al. 2013) resulted in major developmental and energetic defects. In humans, homozygous patents carrying a loss-of-function mutation of MICU1 are characterized by myopathy, cognitive impairment and extrapyramidal movement disorder (Logan et al. 2014), along with an increased agonist-induced mitochondrial Ca^2+^ uptake at low cytosolic Ca^2+^ concentrations and a decreased cytosolic Ca^2+^ signal. However, at least under resting conditions, the fibroblasts from affected individuals do not display defects in overall cellular metabolic function, but chronic elevation of the mitochondrial matrix Ca^2+^ load seems to lead to moderate mitochondrial stress, resulting in fragmentation of the mitochondrial network. In another work, postnatal manipulation of MCU levels in mice (by using adeno-associated virus-mediated gene transfer) demonstrated the contribution of MCUC to the regulation of skeletal muscle tropism. MCU overexpression or downregulation caused muscular hypertrophy or atrophy, respectively, likely independently of metabolic alterations, but dependent on a novel Ca^2+^-dependent mitochondria-to-nucleus signaling pathway via transcriptional regulators (Mammucari et al. 2015). Finally, in mice with myocardial MCU inhibition by transgenic expression of a dominant-negative (DN) MCU, a strong correlation among MCU function, MCU-enhanced oxidative phosphorylation and correct pacemaker cell function has been found (Wu et al. 2015).

Besides its physiological role for muscle function, MCU has also been implicated in cancer-related processes, in particular in the control of metastasis. Recently, Hall and colleagues (Hall et al. 2014) found that breast cancer patient outcomes were negatively correlated with increased MCU Ca^2+^ conducting pore subunit expression and decreased MICU1 regulatory subunit expression. However, they showed that a widely used breast cancer cell line did not require MCU or MICU1 activity for survival in contrast to cervical, colon and prostate cancer-derived cells. Our research in a publically available database suggests that indeed expression of MCU is often altered only slightly in tumoral tissues, in accordance with previous findings (Davis et al. 2013) (Table 2). On the other hand, Tang et al. (2015) revealed that MCU expression correlates with metastasis and invasiveness of breast cancer. MCU inhibition by ruthenium red or MCU silencing by siRNA abolished migration of breast cancer cells and reduced serum- or thapsigargin (TG)-induced store-operated Ca^2+^ entry (SOCE). Serum-induced migrations in these MDA-MB-231 cells were blocked by SOCE inhibitors, suggesting that MCU plays a critical role in breast cancer cell migration by regulating SOCE (Tang et al. 2015). In an independent study, *MCU* expression has been related to breast tumor size and lymph node infiltration. Indeed, in an MDA-MB-231 xenograft model, ablation of MCU induced a reduction in tumor growth and metastasis formation. The mechanism proposed to account for slower tumor progression in MCU-lacking cells envisions reduction in mitochondrial ROS production and via HIF-1alpha and expression of its target genes, in turn inducing a (Tosatto et al. 2016) a decrease in cancer progression genes. In this work, HIF-1alpha has been demonstrated to be a major effector of MCU, since rescuing HIF-1alpha expression the cells restored the tumor cells' ability to migrate. Finally, it has been proposed that a small molecule, AG311, shown to retard tumor growth and to reduce lung metastases, might induce breast cancer cell death by activating MCU, although direct proof is missing (Bastian et al. 2015).

**Table 2:** Oncomine analysis of *CCDC109A* gene in several human cancer tissues. Here are reported only tumors in which gene expression was changed at least twice compare to normal tissues

Gene	Channel	Tissue	Tumor	C vs. N	References
CCDC109A	MCU	Pancreas	Pancreatic carcinoma	3.014	Pei et al. (2009)
				3.577	Iacobuzio-Donahue et al. (2003)
		Kidney	Papillary renal cell carcinoma	2.292	Yusenko et al. (2009)

In summary, there is an urgent need to identify pharmacological agents able to impact mitochondrial calcium uptake via their specific action on MCUC components, since the so-far used Ruthenium Red and lanthanides are wide-spectrum modulators. This task could be much greatly helped by structure-activity relationship (SAR) studies. Unfortunately, only the structure of the N-terminal part of MCU has not been resolved up to now (Lee et al. 2015), and a systematic study linking single-point mutations to channel function is also missing. Nevertheless, this issue is of utmost importance, especially in view of the emerging patho-physiological importance of MCUC.

### Mitochondrial Kv1.3

Kv1.3 is a member of the Shaker family of the potassium channel (Gutman et al. 2005) and is the most expressed channel in the T lymphocytes (Cahalan and Chandy 2009). Plasma membrane Kv1.3 and the other members of the Kv family control the resting and action potential in excitable cells, while in non-excitable tissues it regulates cell volume and proliferation, but also cell death (Armstrong 2003; Gutman et al. 2003; MacKinnon 2003; O’Grady and Lee 2005; Leanza et al. 2014b).

Kv1.3 has been shown to be expressed in brain, lung, thymus, spleen, lymph nodes, fibroblasts, lymphocytes (Szabò et al. 2005), tonsils, macrophages (Leanza et al. 2012b), microglia, oligodendrocytes, osteoclasts, platelets, liver, skeletal muscle, hippocampal neurons (Bednarczyk et al. 2010), astrocytes, and brown and white fat (Gutman et al. 2005; Szabò and Zoratti 2014). Furthermore, Kv1.3 displays an altered expression level in various cancers (Arcangeli et al. 2009; Comes et al. 2015; Bielanska et al. 2009), such as lymphoma (Alizadeh et al. 2000), melanoma (Artym and Petty 2002), glioma (Preussat et al. 2003), breast (Abdul et al. 2003; Jang et al. 2009), prostate (Abdul and Hoosein 2006), gastric (Lan et al. 2005) and colon cancer (Abdul and Hoosein 2002). Kv1.3 was also shown to be present in the mitochondrial inner membrane; in particular, it has been shown to be expressed in the prostate and breast cancer cell lines PC3 and MCF-7, respectively, and lymphoma and leukemia cells in the mitochondria (Leanza et al. 2013b; Szabo et al. 2015; Gulbins et al. 2010).

Results from the Oncomine database are reported in Table 3: Kv1.3 is overexpressed in several tumors that affect different organs, such as the kidney, blood, skin, brain and esophagus, at least two fold with respect to the normal tissues. Changes in Kv1.3 expression in cancer cells have been related to epigenetic mechanisms, such as DNA methylation, as demonstrated in pancreatic cancer (Brevet et al. 2009a) and in poorly differentiated breast cancer (Brevet et al. 2009b).

**Table 3:** Oncomine analysis of *KCNA3 *gene in several human cancer tissues. Here are reported only tumors in which gene expression was changed at least twice compare to normal tissues

Gene	Channel	Tissue	Tumor	C vs. N	References
KCNA3	Kv1.3	Kidney	Chromophobe renal cell carcinoma	4.908	Jones et al. (2005)
			Renal pelvis urothelial carcinoma	4.315	Jones et al. (2005)
		Blood	Chronic lymphocytic leukemia	2.368	Rosenwald et al. (2001)
				3.180	Basso et al. (2005)
				2.197	Haferlach et al. (2010)
			T-cell acute lymphoblastic leukemia	2.198	Haferlach et al. (2010)
			Primary effusion lymphoma	6.402	Basso et al. (2005)
			Mantle cell lymphoma	2.924	Basso et al. (2005)
			Marginal zone B-cell lymphoma	2.701	Storz et al. (2003)
		Skin	Actinic (solar) keratosis	2.667	Nindl et al. (2006)
		Brain	Classic medulloblastoma	2.289	Pomeroy et al. (2002)
		Esophagus	Barrett’s esophagus	2.565	Hao et al. (2006)

Ten years ago, our group discovered the mitochondrial counterpart (mitoKv1.3) of this channel located in the IMM (Szabò et al. 2005). Other members of Kv channels have also been described in mitochondria, i.e., Kv1.5 and Kv1.1 (Vicente et al. 2006; Leanza et al. 2013a). MitoKv1.3 mediates an inward potassium flux to the mitochondrial matrix and has a role in the organellar K^+^ cycle that participates in the modulation of coupling between ATP synthesis and mitochondrial respiration, thus contributing to the regulation of several processes including mitochondrial volume, mitochondrial structural integrity and production of ROS (Szabò et al. 2012). Moreover, mitoKv1.3 has an important role during apoptotic cell death (Szabó et al. 2008). In particular, it has been demonstrated that mitoKv1.3 can interact with pro-apoptotic Bax via a critical lysine (lys128) that protrudes into the mitochondrial intermembrane space (Annis et al. 2005) following translocation of Bax to the outer mitochondrial membrane. Point mutation of this lysine into a glutamic acid led to lack of inhibition of mitoKv1.3 and to a switch in the function of Bax, transforming it into an anti-apoptotic protein (Szabò et al. 2011).

We then provided evidence that pharmacological inhibition of mitoKv1.3 by membrane permeant blockers, the psoralens Psora-4 and PAP-1, and the riminophenazine clofazimine, is sufficient to trigger apoptotic cell death in cancer cells but not in healthy ones (Leanza et al. 2012a, 2013b, 2014a, Szabo et al. 2015). Inhibition of mitoKv1.3 induced a block of the potassium flux into the mitochondria inducing mitochondrial membrane hyperpolarization followed by reduction of the respiratory chain complexes, which causes increased ROS production. ROS can in turn activate both PTP inducing mitochondrial membrane depolarization and favor the detachment of cytochrome c from the cristae and its release in the cytosol activating intrinsic cell death pathway (Leanza et al. 2015b).

Cell death was detected both *in vitro*, even with cancer cells lacking pro-apoptotic Bax and Bak, often downregulated in tumors, and in vivo. In particular, clofazimine reduced the tumor volume by 90 % in an orthotopic mouse melanoma model (Leanza et al. 2012a). Furthermore, these compounds acted also on primary tumor B-cells obtained from patients affected by chronic lymphocytic leukemia (CLL), one of the most diffuse forms of leukemia in the Western world. Indeed, in *ex*-in vivo experiments, pathological B-cells underwent cell death while healthy cells from the same patients did not. Importantly, these effects were also obtained with the B-CLL cells that are mutated in p53, or overexpressing anti-apoptotic Bcl-2, and were independent of the currently used prognostic factors (ZAP70, CD38 and hypersomatic mutation) (Leanza et al. 2013b). Moreover, these compounds killed primary tumor B-cells even when they were co-cultured together with mesenchymal stromal cells, which mimic the lymph node micro-environment, favoring the pathological B-cells' survival (Szabo et al. 2015). The selectivity versus cancer cells, according to our experiment-based model, is due to a synergistic effect of mitoKv1.3 inhibition and an increase of ROS: reactive oxygen species are often altered in tumor cells leading them to more easily reach the critical threshold necessary to trigger cell death upon exogenous oxidative insult (Ralph et al. 2010). In contrast, normal cells, even if an increased ROS production is induced, do not reach the threshold and do not undergo apoptosis (Trachootham et al. 2009). Importantly, the fact that clofazimine is a drug that is already used in the clinic to treat leprosy and some autoimmune diseases with a very good safety profile (Cholo et al. 2012) leads to the possibility for drug repositioning, i.e., for treating Kv1.3-expressing tumors with this drug.

Interestingly, it has recently been demonstrated that Kv1.3 channels are localized in the nucleus of several types of cancer cells and human brain tissues where they are able to regulate the nuclear membrane potential and activation of transcription factors, such as phosphorylated CREB and c-Fos (Jang et al. 2015). Furthermore, Kv1.3 has been shown to be present also in the cis-Golgi, even if its role in this intracellular membrane and the eventual connection with cancer development have not been investigated yet (Zhu et al. 2014).

### Mitochondrial BKCa (KCa1.1)

The large-conductance calcium- and voltage-activated K^+^ channel BKCa (KCa1.1) is expressed at the plasma membrane of both excitable and non-excitable cells, including sensory and epithelial cells. It is also expressed in the smooth muscle and cardiac muscle, where it is involved in muscle contraction but also in cytoprotection during ischemia/reperfusion. Furthermore, plasma membrane BKCa also has a role in hypertension and cancer cell metastasis (Cui et al. 2009; Sah 1996; Eichhorn and Dobrev 2007; Félétou 2009; Sokolowski et al. 2011). BKCa has also been revealed in intracellular membranes, such as the nuclear membrane, ER, Golgi and mitochondria (Singh et al. 2012). The existence of mitoBKCa has been proven by electrophysiological experiments, as well as by Western blotting, electron microscopy and immunofluorescence (O’Rourke 2007; Szewczyk et al. 2010; Douglas et al. 2006; Kathiresan et al. 2009; Piwonska et al. 2008; Skalska et al. 2008). MitoBKCa has been observed in the mitochondria of glioma cell lines, astrocytes as well as ventricular cells, skeletal muscle, brain and endothelial cells. The known modulators of the plasma membrane channel also act on the mitoBKCa. These compounds can be divided into activators, such as calcium, diCl-DHAA (Sakamoto et al. 2008), NS1619 (Skalska et al. 2009), 17-estradiol (Ohya et al. 2005) and hypoxia (Cheng et al. 2008), or inhibitors, such as charybdotoxin (Gu et al. 2007; Skalska et al. 2008), iberiotoxin (Cheng et al. 2008, 2011) and paxillin (Heinen et al. 2007a, b), but no drug acting exclusively on the mitochondrial channel is available up to now.

The role of mitoBKCa in pathophysiological conditions seems to consist of the modulation of mitochondrial Ca^2+^ uptake. Indeed, its opening protects against damage to the heart and other organs caused by ischemia and reperfusion, possibly by preventing calcium overload (O’Rourke 2007; Xu et al. 2002). mitoBKCa has also been associated with cell death, since Bax can inhibit the channel leading to the activation of the MPTP (Cheng et al. 2011). To date, no evidence about a possible role of mitoBKCa in cancer development has been reported, except for a possible role in glioma cell motility after irradiation: BK channel activity is augmented by increasing the open probability but not the number of the channels, which results in activation of CaMKII leading to enhanced glioblastoma cell migration (Steinle et al. 2011).

### Mitochondrial IKCa (KCa3.1)

The intermediate-conductance potassium channel (IKCa or KCa3.1) is expressed in various tissues, such as epithelial and endothelial tissues, immune system, sensory neurons and microglia but not in excitable tissues. The IKCa is involved in several physiological processes modulating membrane potential and calcium signaling, including cell proliferation and differentiation in numerous cell types (Szabò and Zoratti 2014). The most known and used blockers are TRAM-34, cyclohexadiene 4 and clotrimazole (Wulff and Castle 2010). IKCa showed a different expression in cancer with respect to normal cells: a possible explanation for this variation could be ascribed to changes at the transcriptional level. The RE1-silencing zinc-finger transcription factor (REST) binds to a 21-bp DNA element (RE1) within the regulatory region of its target genes, repressing gene expression. REST expression can be abnormally regulated in cancer cells (e.g., Shimojo et al. 2013). There are more than 1300 genes that contain an RE1 element, including genes that encode proteins of fundamental importance for cell function such as ion channels. A reduced expression of REST can result in the transcriptional activation of KCa3.1, as observed in vascular cells (Cheong et al. 2005). Mutations that compromise REST transcriptional repression have been associated with different cancers, e.g., with childhood renal cancer (Mahamdallie et al. 2015). Thus, one intriguing possibility is that expression of ion channels in general, including that of IKCa in cancer cells, is linked to regulation by functionally altered REST.

An intracellular localization for the IKCa (mitoIKCa) in the inner mitochondrial membrane of human colon carcinoma and HeLa cells, as well as in mouse embryonic fibroblasts, has been demonstrated by electrophysiology and Western blotting (De Marchi et al. 2009; Sassi et al. 2010). The plasma membrane channel and mitochondrial one seem to have the same structure and physiological proprieties, since also the mitoIKCa is inhibited by TRAM-34 and clotrimazole.

The role of mitoIKCa in the IMM has not been investigated in detail, but it is expected to be similar to the one hypothesized for the mitoKv1.3 and other mitochondrial potassium channels, i.e., a contribution to the regulation of the organelle membrane potential, volume and ROS production. A possible role for the mitoIKCa was also postulated during cell death, even if this channel, contrarily to mitoBKCa and mitoKv1.3, did not interact with Bax. Nevertheless, a possible role of mitoIKCa in inducing cell death has to be verified, since a connection between IKCa channels and the intrinsic apoptotic pathway has been observed (McFerrin et al. 2012). Furthermore, while TRAM-34 alone was not able to induce cell death when used at the micromolar range, it increased the sensitivity of melanoma cells to TRAIL treatment (Quast et al. 2012).

### Mitochondrial TWIK-related acid-sensitive K^+^ channel-3 (TASK-3)

The two-pore K^+^ (K2P) channels, to which the Tandem of P-domains weakly inward rectifying K^+^ (TWIK)-related acid sensitive K^+^ channel 3 (TASK-3) belongs, is the most recently identified group among the K^+^ channels. K2P channels are ‘leak’ K^+^ channels that set the resting membrane potential and regulate cell excitability (Felipe et al. 2006; Bayliss and Barrett 2008). TASK-3 is normally found in the adrenal cortex, gastrointestinal tract, neuronal tissue and salivary glands (Bayliss and Barrett 2008; Kovacs et al. 2005) and is modulated by alterations in extracellular pH and by anaesthetic agents; it plays a role in aldosterone secretion (Bayliss and Barrett 2008; Bayliss et al. 2003; Ekhterae et al. 2003; Patel and Lazdunski 2004). Altered TASK-3 expression has already been defined in several types of cancer, i.e., breast cancer, and the gene encoding TASK-3 (KCNK9) was found to be overexpressed by 5- to >100-fold in 44 % of tumors (Mu et al. 2003). TASK-3 expression has also been identified in mitochondria of melanoma, keratinocytes (Rusznák et al. 2008) and healthy intestinal epithelial cells (Kovacs et al. 2005). Furthermore, this channel is also expressed in lung, colon and ovarian cancers (Felipe et al. 2006; Kim et al. APMIS 2004; Pocsai et al. 2006; Innamaa et al. 2013). Silencing the expression of TASK-3 resulted in compromised mitochondrial function, i.e., mitochondrial membrane depolarization, and reduced cell survival inducing apoptotic cell death in WM35 and A2058 melanoma cells (Kosztka et al. 2011; Nagy et al. 2014). Two aspects linked TASK-3 to cancer development or treatment: (1) observed migration and invasion-reducing effects of TASK-3 overexpression in breast cancer cells (Lee et al. 2012) and (2) increased apoptosis induced by TASK-3 blockers (zinc and methanandamide) in ovarian carcinoma (Innamaa et al. 2013). Further investigation will be necessary to understand whether these effects are related to the mitochondrial channel expression and whether it is possible to pharmacologically regulate mtTASK-3 causing tumor cell death. Since no highly specific mtTASK-3 modulators are available, this aim is difficult to investigate, and the only advance in this field is represented by dihydropyrrolo[2,1-α]isoquinolines (DPIs) compounds, which are able to inhibit TASK channels and could be possible candidates for developing new specific inhibitors (Noriega-Navarro et al. 2014).

### Mitochondrial magnesium channel Mrs2

Mitochondria not only store calcium, but also take up magnesium to maintain the optimal cytosolic Mg^2+^ concentration (0.5–0.7 mM) (Kubota et al. 2005). Mg^2+^ can be accumulated inside the mitochondria via the Mg^2+^-selective channel of the inner mitochondrial membrane (Kolisek et al. 2003), which takes advantage of the driving force produced by the mitochondrial membrane potential and is feedback regulated by the increasing Mg^2+^ concentration in the matrix (Khan et al. 2010). An early increase in cytosolic Mg^2+^, which also favors cytochrome c release, occurs during apoptotic cell death (Chien et al. 1999; Kim et al. 2000). Moreover, knockdown of the mitochondrial magnesium channel (Mrs2) caused cell death by inducing loss of respiratory complex I and mitochondrial membrane depolarization (Piskacek et al. 2009). On the contrary, an upregulation of Mrs2 has been observed in parental human gastric adenocarcinoma cell lines, indicating that high expression of Mrs2 may protect against death (Chen et al. 2009; Wolf and Trapani 2009). In agreement, an augmented expression of the *mrs2* gene has been reported by comparing normal and cancer organs as shown in Table 4: a general increase of around two-three-fold (but in testis tumor a 17-fold increase) has been observed in blood, skin, ovarian, kidney, breast, lung and bladder tumors when compared to the normal tissues, meaning that it would be worthwhile to further deepen our understanding of the possible role of mitochondrial Mg^2+^ fluxes in cancer development (see Table 4 and the references therein).

**Table 4:** Oncomine analysis of *MRS2* gene in several human cancer tissues. Here are reported only tumors in which gene expression was changed at least twice compare to normal tissues

Gene	Channel	Tissue	Tumor	C vs. N	References
MRS2	Mrs2	Testis	Testicular embryonal carcinoma	17.178	Skotheim et al. (2005)
				9.762	Korkola et al. (2006)
			Testicular seminoma	3.812	Skotheim et al. (2005)
				3.281	Korkola et al. (2006)
			Testicular intratubular germ cell neoplasia	2.754	Skotheim et al. (2005)
			Testicular yolk sac tumor	2.213	Skotheim et al. (2005)
				4.199	Korkola et al. (2006)
			Testicular teratoma	2.006	Skotheim et al. (2005)
		Skin	Skin basal cell carcinoma	3.487	Riker et al. (2008)
			Cutaneous melanoma	3.469	Riker et al. (2008)
			Skin squamous cell carcinoma	2.056	Riker et al. (2008)
		Ovarian	Ovarian serous adenocarcinoma	3.105	Yoshihara et al. (2009)
		Kidney	Papillary renal cell carcinoma	3.070	Yusenko et al. (2009)
			Chromophobe renal cell carcinoma	2.283	Yusenko et al. (2009)
		Breast	Ductal breast carcinoma	2.775	Richardson et al. (2006)
			Ductal breast carcinoma in situ	2.049	Ma et al. (2009)
			Invasive breast carcinoma	2.376	Finak et al. (2008)
		Blood	T-cell childhood acute lymphoblastic leukemia	2.517	Coustan-Smith et al. (2011)
			B-cell childhood acute lymphoblastic leukemia	2.036	Coustan-Smith et al. (2011)
			Anaplastic large cell lymphoma	2.400	Piccaluga et al. (2007)
			Angioimmunoblastic T-cell lymphoma	2.023	Piccaluga et al. (2007)
		Lung	Pleural malignant mesothelioma	2.458	Gordon et al. (2005)
		Bladder	Superficial bladder cancer	2.440	Dyrskjøt et al. (2004)
			Infiltrating bladder urothelial carcinoma	2.073	Dyrskjøt et al. (2004)

### Mitochondrial uncoupling protein UCP

The Uncoupling protein (UCP) family is constituted by five members and belongs to the mitochondrial anion-carrier proteins (Krauss et al. 2005). UCPs are inner mitochondrial membrane proteins involved in the re-entry of protons into the mitochondrial matrix, partially dissipating the electrochemical gradient and therefore the mitochondrial membrane potential. As a consequence, UCP2 regulates mitochondrial ROS production as well. UCP-2 is ubiquitously expressed in different tissues, including skeletal muscle and β-cells, and it is overexpressed in numerous tumors, such as breast, ovarian, bladder, esophagus, testicular, colorectal, kidney, pancreatic, lung and prostate cancers and leukemia (Leanza et al. 2014b).

UCP2 has been proposed to impact cell survival by decreasing the formation of mitochondrial superoxide production by tuning the proton leak and thus the mitochondrial membrane potential (Baffy 2010; Baffy et al. 2011; Basu Ball et al. 2011; Deniaud et al. 2012). Indeed, UCP2 overexpression protects against ROS production and increases the apoptotic threshold for cancer cell survival (Zhang et al. 2007), while UCP2 knockdown or pharmacological inhibition leads to an increase in mitochondrial ROS, as observed in UCP2 knockout mice (Arsenijevic et al. 2000). Moreover, UCP2 downregulation or inhibition triggers an ROS-mediated autophagy in pancreatic adenocarcinoma cells (Dando et al. 2013).

UCP2 overexpression has been observed after oxidative stress induced by respiratory chain inhibitors (Giardina et al. 2008) and has been related to the development of breast cancer in an orthotopic model (Ayyasamy et al. 2011). Furthermore, UCP-2 overexpression was correlated with the Warburg phenotype (Samudio et al. 2008). Additionally, UCP2 was proposed to catalyze an exchange of malate, oxaloacetate and aspartate for phosphate; it exports C4 metabolites from mitochondria to the cytosol in vivo, providing evidence that UCP2 reduces mitochondrial oxidation of glucose and enhances glutaminolysis. These results postulate a unique regulatory mechanism in cell bioenergetics and explain the significance of UCP2 levels in metabolic reprogramming occurring under various physio-pathological conditions (Vozza et al. 2014).

Importantly, UCP2 overexpression prevented the death-inducing effect of chemotherapy in different cancer cell lines: indeed, treatment with gemcitabine stimulates UCP2 mRNA production, suggesting a role of mitochondrial uncoupling in the resistance to this chemotherapeutic agent, and pointed out the possible synergistic antiproliferative effect of coupling gemcitabine treatment and UCP2 inhibition (Derdak et al. 2008; Dalla Pozza et al. 2012; Yu et al. 2015a). Onconase, a member of the RNase super-family, is able to inhibit UCP2 and manganese-dependent superoxide dismutase (MnSOD), thus triggering mitochondrial superoxide ion production leading to autophagy (Fiorini et al. 2015). Similarly, a decrease in cell viability and clonogenicity, in addition to an increase in ΔΨm, ROS production, apoptosis and autophagy, was induced in breast cancer cells after both UCP2 inhibition by siRNA and cytotoxic treatments by tamoxifen (Gabriel Pons et al. 2015). Likewise, MiR-214 sensitizes breast cancer cells to both tamoxifen and fulvestrant treatment by targeting UCP2 (Yu et al. 2015b). Finally, UCP3 has been linked to clear cell renal cell carcinoma: it has been proposed that inhibition of UCP3 by ADP might contribute to the setting of the endogenous mitochondrial membrane potential (Lim et al. 2015).

In summary, modulation of oxidative stress in cancer cells is a powerful tool to kill or at least sensitize them to chemotherapeutic treatments, as has been observed with mitoKv1.3. In this scenario, a primary role could be attributed to UCP proteins, especially to UCP2 and UCP3.

## Intracellular chloride channel CLIC-4

An emerging class of chloride-permeable channels involved in cancer development is the intracellular chloride channels (CLICs). In particular, chloride intracellular channel 4 (CLIC4) is the most well-characterized member of a family of channel proteins that is highly conserved from *Caenorhabditis elegans* to humans. The intracellular CLIC4/mtCLIC has both soluble and membrane-inserted forms and can be localized in different subcellular compartments, such as the mitochondrial inner membrane (Fernández-Salas et al. 1999), cytoplasm, ER membrane, and nucleus. The expression of the CLIC4 transcript is regulated by p53 and tumor necrosis factor α (TNFα), and *clic4* is a direct response gene for both c-myc and p53, two mediators of cancer pathogenesis in multiple tumor sites (Peretti et al. 2015).

CLIC4 overexpression induced apoptotic cell death mediated by loss of the mitochondrial membrane potential, cytochrome c release and caspase activation (Fernández-Salas et al. 2002). On the other hand, inhibition of CLIC4 expression triggered mitochondrial apoptosis under starvation and enhanced autophagy in human glioma cells (Zhong et al. 2012; Xu et al. 2013). Marked changes in expression and subcellular localization of CLIC4 occur early in tumorigenesis: (1) CLIC4 expression was reported to be a marker of colon cancer stem cells and is associated with a poor prognosis, (Deng et al. 2014); (2) reduced CLIC4 expression and nuclear translocation, following cellular stress conditions, e.g., DNA damage, senescence and metabolic alterations, is associated with the altered redox state, and CLIC4 acts as an important suppressor of cutaneous squamous cell cancer development and progression (Suh et al. 2012); (3) KRAS-mediated downregulation of CLIC4 may promote cancer carcinogenesis in a specific fraction of lung cancers (Okudela et al. 2014). Moreover, circulating CLIC4 was identified as a biomarker for epithelial ovarian cancer (Tang et al. 2013). Thus, in addition to an important function in cancer pathogenesis as a tumor suppressor, CLIC4 is emerging as a potential biomarker to monitor tumor progression and recurrence in multiple human cancers.

## Intracellular potassium channel Kv10.1

A surprising localization of the human ether à-go-go1 protein (Eag1 or Kv10.1), a potassium voltage-gated channel subfamily H member 1, has been described in the nuclear inner membrane (Chen et al. 2011). There, the channel seems to be involved in setting the nuclear K^+^ concentration thereby affecting gene expression, as postulated also for the nuclear Kv1.3. Even if expressed in the plasma membrane, Kv10.1 is also rapidly internalized to lysosomes (Kohl et al. 2011), as demonstrated also by patch clamp measures (Wang et al. 2012).

An increased expression of Kv10.1 can be triggered by the tumor suppressor p53, as observed in glioblastoma (Bai et al. 2013), and the transcription factor E2F1 (Lin et al. 2011). Overexpressed Kv10.1 controls cancer cell migration and proliferation by interactions with RAB proteins (Ninkovic et al. 2012), cortactin (CTTN) and focal adhesion kinase (FAK) (Herrmann et al. 2012) as well as through calcium signaling (Hammadi et al. 2012) and an altered response to hypoxia (Downie et al. 2008). Overexpression by genomic amplification has been implicated as a mechanism of Kv10.1 involvement in a small proportion of colon (3.4 %) and head and neck (15 %) cancers (Pardo and Stühmer 2014).

Eag1 is not detectable in healthy tissues except the brain, but its overexpression, not discriminating between the plasma membrane and intracellular channels, has been detected at a very high rate (>75 %) in several tumors: breast, renal and cervical carcinoma cell lines, as well as in different human malignancies, for instance, colorectal and cervical cancer, soft tissue sarcomas, acute myeloid leukemia, esophageal and gastric cancer, head and neck carcinomas, ovarian, breast, lung and prostate cancer and glioblastoma (Martínez et al. 2015 and references therein). Importantly, Kv10.1 expression correlates with poor prognosis in fibrosarcoma, ovarian carcinoma, acute myeloid leukemia (AML) and colon, head and neck cancer (Pardo and Stühmer 2014).

Furthermore, Kv10.1 transfection in healthy cells induces transformation and leads the transfected cells to develop invasive tumors in vivo: this was the first observation of the oncogenic capacity of a potassium channel (Pardo et al. 1999). Interestingly, a Kv10.1 mutant that lacks potassium permeability reduces, but does not abolish tumorigenesis (Downie et al. 2008; Hegle et al. 2006). Despite all these observations, the exact contribution of the intracellular Eag1 to cancer cell survival remains to be determined.

## Intracellular Transient Receptor Potential Channel (TRPs)

The transient receptor potential (TRP) channel superfamily is one of the largest families of cation channels (Nilius and Owsianik 2011). The TRP family is constituted by 28 members, divided into subfamilies, which are TRPC (canonical), TRPM (melastatin), TRPP (polycystin), TRPV (vanilloid), TRPML (mucolipin) and TRPA (ankyrin-like) (Nilius 2007). Among them, the intracellularly located channels that are related to cancer development and progression identified so far are TRPM8 and TRPC1.

The calcium-permeable TRPM8 is located in the endoplasmatic reticulum (ER) membrane and is overexpressed in several tumors (Zhang and Barritt 2004). TRPM8 trafficking is mediated during carcinogenesis by interaction and consequent regulation by TRPM8 channel-associated factor 1 and 2 (TCAF-1 and TCAF-2). In particular, TCAF-1 is more expressed in the early tumor stages favoring cancer development, while its expression as well as that of TRPM8 is reduced during the tumor spreading with metastasis (Gkika et al. 2015). Intracellular TRPM8 mediates the decrease in Ca^2+^ concentration inside the ER and favors the resistance to apoptotic cell death (Bidaux et al. 2007). As mentioned above, an increase in intracellular Ca^2+^ leads to modulation of the signaling pathways and to transcription of genes that mediate the cellular responses to mitogens and chemoattractants.

TRPM8 expression in normal conditions is tissue-selective, while when TRPM8 is abnormally expressed, it has been observed in several different cancer tissues, such as prostatic, pancreatic, breast and colorectal ones, in urinary bladder and oral squamous cells, and in lung carcinoma, melanoma, neuroblastoma and osteosarcoma (Yee 2015). In particular, expression of TRPM8 in breast carcinoma correlates with the histological grade, Ki-67, tumor size and expression of the estrogen receptor. These findings suggest that TRPM8 channels play a pivotal role in the development and growth of mammary tumors (Dhennin-Duthille et al. 2011; Chodon et al. 2012; Liu et al. 2014). Experimental data demonstrated that TRPM8 channels play important roles in general in cancer cells, in particular in their proliferation, survival, migration, invasion and neurosecretion. For example, the regulation of proliferation in cancer cell lines has been proven by siRNA experiments, in which downregulation of TRPM8 caused the reduction of prostatic and osteosarcoma cancer cell proliferation and cell cycle progression (Valero et al. 2012; Wang et al. 2014). On the other hand, in lung carcinoma, the ectopic expression of TRPM8 favors cell propagation (Du et al. 2014). These data suggest a tumor-specific role of TRPM8 in proliferation. Furthermore, several in vivo studies have linked TRPM8 to cancer growth and metastasis (Yee 2015). The aberrant overexpression of TRPM8 in tumors and its proliferative and invasive roles suggest a future development of the TRPM8 channel as a prognostic/predictive biomarker and a therapeutic target in oncology.

Another member of this superfamily located in the ER is TRPC1. This was the first and most studied member in the context of cancer, being involved in several features of cancer cells, such as cell fate and motility (Shapovalov et al. 2016). This channel has a specific regulation in cancer, also depending on the stage of malignant transformation: the TRPC1 expression level decreases with the progression of the prostate cancer from the androgen-dependent to androgenin dependent phase (Nilius et al. 2007). The role of TRPC1 in cancer progression is predominantly related to cell motility. In particular, inhibition of expression and/or activity in nasopharyngeal carcinoma led to reduced adhesion and invasiveness of cancer cells (He et al. 2012). Furthermore, pharmacological inhibitors, such as SKF96365, 2-APB and MRS1845, or siRNA against TRPC1 was able to suppress human malignant glioma proliferation (Bomben and Sontheimer 2010). Similar effects have also been found in a lung carcinoma cell line, in which TRPC1 ablation by siRNA induced blocking of cell growth due to G0/G1 cell cycle arrest (Bomben et al. 2011).

## Intracellular IP3 receptors

The two major families of Ca^2+^ channels involved in the release of this cation from the ER include the inositol 1,4,5-trisphosphate receptors (IP3Rs) and ryanodine receptors (RyRs). In cancer cells, Ca^2+^ mobilization has been observed to contribute to tumor progression, and increasing numbers of studies indicate the relevance of IP3Rs to cancer. (Prevarskaya et al. 2011; Roderick and Cook 2008; Lee et al. 2011; Monteith et al. 2012). Indeed, type 1 IP3R (i.e., IP3R1) expression is reduced and type 3 IP3R (i.e., IP3R3) expression is increased in human glioblastoma tissues compared to normal human brain, and inhibition of IP3R3 by caffeine reduces the migration, invasion and survival of glioblastoma cells (Kang et al. 2010). Similar effects as the one induced by caffeine have been observed in breast cancer cells in the presence of 17β-estradiol (E2) (Szatkowski et al. 2010). Increased IP3R3 expression was also related to aggressiveness in colorectal cancer: high levels of IP3R were associated with increased metastasis in the lymph nodes and liver and a decreased 5-year survival (Shibao et al. 2010). Moreover, changes in IP3R1 have been implicated in the biological properties of the tumors: for example, cisplatin-induced downregulation of IP3R1 expression was found to be associated with the acquisition of cisplatin resistance in bladder cancer cell lines (Tsunoda et al. 2005). Furthermore, Bcl-2 family proteins, which have pro- and anti-apoptotic functions, directly bind to different sites on IP3Rs and impact IP3R function, suggesting that IP3R is an important hub for the action of Bcl-2 family proteins in various physiological and pathological settings including tumor progression (Seo et al. 2015; Oakes et al. 2005; Letai 2008). In addition, other proto-oncogenes as well as tumor suppressors, such as Akt/protein kinase B (PKB) (Chan and Tsichlis 2001), Bax inhibitor-1 (BI-1) (Li et al. 2007a; Eckenrode et al. 2010) and K-ras-induced actin-interacting protein (KRAP) (Fujimoto et al. 2011), modulate cancer development by acting on Ca^2+^ fluxes mediated by the IP3Rs (Akl and Bultynck 2013). Currently, little is known about what specific players of Ca^2+^ signaling contribute to the altered Ca^2+^ mobilization in cancer cells and how Ca^2+^ signaling cross-talks with well-known cancer pathways, such as the Ras/Raf/MAPK pathway (Dhillon et al. 2007). Recently, a paper has connected the Ras signaling pathway, often deregulated in cancer (Downward 2003), to the IP3R-mediated Ca^2+^ release pathway. Indeed, oncogenic K-Ras inhibited IP3-induced Ca^2+^ release from the ER by remodeling of IP3R isoform composition in a human colorectal cancer cell line harboring the K-Ras mutant allele (G13D) (Pierro et al. 2014). A further link between K-Ras and IP3R is suggested by the finding that K—the GTP-bound active form of K-Ras4B—forms a ternary complex with IP3R and Bcl-xL and promotes cell death, indicating that IP3R is a novel effector of K-Ras4B. (Sung et al. 2013).

Finally, IP3Rs have also been shown to interact with other ion channels. Indeed, IP3R3 is able to favor breast cancer cell proliferation by co-localizing and directly interacting with BKCa channels, but only in cancerous cells and not in the healthy ones (Mound et al. 2013).
